# Influence of *UGT1A1* Genetic Variants on Free Bilirubin Levels in Japanese Newborns: A Preliminary Study

**DOI:** 10.3390/ijerph192013090

**Published:** 2022-10-12

**Authors:** Hiroaki Hanafusa, Shinya Abe, Shohei Ohyama, Yuki Kyono, Takumi Kido, Ruka Nakasone, Mariko Ashina, Kenji Tanimura, Kandai Nozu, Kazumichi Fujioka

**Affiliations:** 1 Department of Pediatrics, Kobe University Graduate School of Medicine, Kobe 650-0017, Japan; 2 Department of Obstetrics and Gynecology, Kobe University Graduate School of Medicine, Kobe 650-0017, Japan

**Keywords:** free bilirubin, bilirubin encephalopathy, genetic variants, hyper-free bilirubinemia, uridine diphosphate glucuronosyltransferase

## Abstract

Background: Free bilirubin (Bf) is a better marker than total serum bilirubin (TSB) for predicting bilirubin encephalopathy (BE). To date, two *UGT1A1* genetic variants (rs4148323 and rs3064744) have been associated with neonatal hyperbilirubinemia; however, the direct association between *UGT1A1* variants and Bf levels in newborns has not been elucidated. Methods: We retrospectively analyzed the clinical data of 484 infants, including the genotype data of two *UGT1A1* genetic variants. We divided the infants into a high Bf group (Bf ≥ 1.0 µg/dL, *n* = 77) and a non-high Bf group (Bf < 1.0 µg/dL, *n* = 407), based on the peak Bf values. Logistic regression analysis was performed to calculate the odds ratios (ORs) for each variant allele compared to wild-type alleles. Results: The frequencies of the A allele in rs4148323 and (TA)_7_ allele in rs3064744 in the high Bf group (29% and 4%, respectively) were significantly different from those in the non-high Bf group (16% and 12%, respectively). In logistic regression analysis, for rs4148323, the A allele was significantly associated with an increased risk of hyper-free bilirubinemia over the G allele (adjusted OR: 1.80, 95% confidence interval [CI]: 1.19–2.72, *p* < 0.01). However, for rs3064744, the (TA)_7_ allele was significantly associated with a decreased risk of hyper-free bilirubinemia over the (TA)_6_ allele (adjusted OR: 0.42, 95% CI: 0.18–0.95, *p* = 0.04). Conclusions: This study is the first to show that the A allele in rs4148323 is a risk factor and that the (TA)_7_ allele in rs3064744 is a protective factor for developing hyper-free bilirubinemia in Japanese newborns.

## 1. Introduction

Newborns are prone to hemolysis due to their physiological polycythemia and short fetal hemoglobin life. In addition, the glucuronidation activity in neonates is low, resulting in the development of physiological jaundice, which is characterized by indirect bilirubin-dominant hyperbilirubinemia. Neonatal jaundice does not require treatment when the bilirubin level is in its physiological range, but hyperbilirubinemia can cause bilirubin encephalopathy [1]. Although neonatal jaundice has been managed based on total serum bilirubin (TSB) levels for decades, it has been suggested that even relatively mild hyperbilirubinemia can cause various neurological sequelae [2,3]. Regarding bilirubin neurotoxicity, the transfer of bilirubin to the central nervous system was reported to be suppressed by albumin (Alb) co-administration in an animal model [4]. In addition, it was also shown that most of the bilirubin deposited in the brain was not bound to Alb [5]. Thus, researchers, including the authors, have indicated that free bilirubin (Bf), that is, indirect bilirubin not bound to Alb, is a more important marker for predicting bilirubin encephalopathy because it crosses the blood–brain barrier [6,7,8,9]. In recent years, it has been reported that, when TB is 205.2 μmol/L (12 mg/dL) or more, the correlation between Bf and TB is no longer observed, and there is a risk of unexpected hyper-free bilirubinemia [10], and 39% of preterm infants with bilirubin encephalopathy did not show hyper-total bilirubinemia but only hyper-free bilirubinemia [11]. Thus, we believe that neonatal jaundice management using Bf measurement is indispensable for the prevention of bilirubin encephalopathy [12,13].

Glucuronic acid conjugation or glucuronidation is a mechanism for making highly lipophilic compounds, such as bilirubin, water-soluble for excretion from the body. This process is catalyzed by uridine diphosphate glucuronosyltransferase (UDP-glucuronosyltransferase). Among the many known isoforms of UDP-glucuronosyltransferase, UDP-glucuronosyltransferase 1A1 encoded by *UGT1A1* (NM_000463.2) catalyzes the glucuronidation of bilirubin [14]. To date, there have been many reports of the association between *UGT1A1* genetic variants and neonatal hyper-total bilirubinemia. Yang et al. investigated the contribution of 11 common mutations and polymorphisms across five bilirubin metabolism genes, including *UGT1A1,* to significant neonatal hyper-total bilirubinemia in the Chinese population. They reported that the presence of c.211G>A of *UGT1A1* (rs4148323) was correlated with an increased risk of hyper-total bilirubinemia, whereas the presence of (TA)_7_ in the TATAA element of the promoter of *UGT1A1* (rs3064744) was correlated with a decreased risk of hyper-total bilirubinemia [15]. Regarding rs4148323, similar results have also been reported from Taiwan [16], the Guangxi area in China [17], Singapore [18], and Japan [19]; therefore, rs4148323 is considered the main genetic contributor for pathological neonatal jaundice in the Asian region. Regarding rs3064744, there have been several conflicting reports that it is a protective factor for hyper-total bilirubinemia in Japan [20] and China [21], whereas it is reported to be a risk factor for hyper-total bilirubinemia in India [22] and Turkey [23]; thus, the effect of this variant on neonatal hyper-total bilirubinemia remains controversial.

Therefore, it is well accepted that these specific variants of *UGT1A1* are involved in determining serum TSB levels in Asian populations. In fact, in addition to reports that Asian newborns are more likely to develop neonatal jaundice [24], there are studies showing that Asian races have higher TSB levels than other races, even in adulthood [25]. However, there was no report of kernicterus in a 10-year national surveillance study in Singapore [26]. In addition, the incidence of kernicterus was reported as 0.86 per 100,000 live births in Taiwan [27], which is not significantly higher than that in Western countries, for example, 1.3 per 100,000 live births in Sweden [28]. Thus, the contribution of *UGT1A1* genetic variants to kernicterus or bilirubin encephalopathy has not been elucidated. Further, it is unclear whether these variants are directly involved in the determination of Bf levels in newborns.

Thus, this study aimed to investigate the association between genetic variants of *UGT1A1* and hyper-free bilirubinemia, which is more directly associated with the development of bilirubin encephalopathy than hyper-total bilirubinemia.

## 2. Materials and Methods

### 2.1. Patients and Samples

We retrospectively analyzed infants who were admitted to Kobe University Hospital between 1 April 2010 and 31 December 2018, whose clinical, laboratory, and *UGT1A1* variant data were available. We obtained clinical data, including gestational age, birth weight, sex, and Apgar scores, and laboratory data, including Bf, TB, DB, Alb levels, and genotype data of rs4148323 and rs3064744 of *UGT1A1.* The values of TB, DB, and Alb were adopted at the time of peak Bf levels. Infants with genetic or chromosomal abnormalities were excluded. In addition, we excluded infants whose DB levels were ≥17.1 μmol/L (1.0 mg/dL), since it was suggested that Bf values determined by UB analyzer (Arrows, Osaka, Japan) were inaccurate when the DB values exceeded this value [29].

As a preliminary study, we investigated the genetic variants of UGT1A1 using residual blood samples collected for clinical laboratory examination. To determine *UGT1A1* variants, the Invader^®^ UGT1A1 Molecular Assay (Sekisui Medical Co., Ltd., Tokyo, Japan) was used, which requires 2.0 mL of whole blood. Serum Bf levels were measured using an automated UB Analyzer (Arrows) via the glucose oxidase–peroxidase (GOD-POD) method, as previously described [30,31]. The TB, DB, and Alb levels were measured using spectrophotometry, the bilirubin oxidase method, and the modified bromocresol purple method, respectively.

As a routine practice in our institution, infants are screened daily via transcutaneous bilirubin level measurement, and, when the transcutaneous bilirubin level exceeds the hour-specific threshold level, serum TB and Bf levels are measured by blood sampling. Following our treatment criteria, infants received phototherapy based on TB or Bf threshold levels stratified by postconceptional age [9,12]. Based on our previous evidence [9,32], we divided the enrolled patients into high (Bf ≥ 1.0 µg/dL) and non-high Bf groups (Bf < 1.0 µg/dL), based on their peak Bf values.

### 2.2. Statistical Analyses

Data are described as median (range) or numbers (%), and OR (95% CI). Mann–Whitney nonparametric rank, Fisher’s exact test, and Chi-square test were used to compare the two groups. Hardy–Weinberg equilibrium was tested for both of the variants with Chi-square test. To determine the effect of the *UGT1A1* variants on hyper-free bilirubinemia, a logistic regression model was used to calculate the ORs (95% CI) for each of the heterozygote allele carriers, homozygote allele carriers, and heterozygote or homozygote carriers to wild-type carriers and to calculate the ORs (95% CI) for each of the variant alleles to wild-type alleles. Multivariate logistic regression analysis was performed to calculate the adjusted odds ratio (OR). Based on previously published evidence [13,16,33,34], the following variables were examined as potential confounders influencing the development of hyper-free bilirubinemia: gestational age, sex, albumin levels, neonatal asphyxia (Apgar score < 7 at 1 min), and other *UGT1A1* variants. 

Statistical analyses were performed using EZR (Saitama Medical Center, Jichi Medical University), a graphical user interface for R (R Foundation for Statistical Computing). Statistical significance was set at *p* value < 0.05. 

## 3. Results

**Figure 1:**
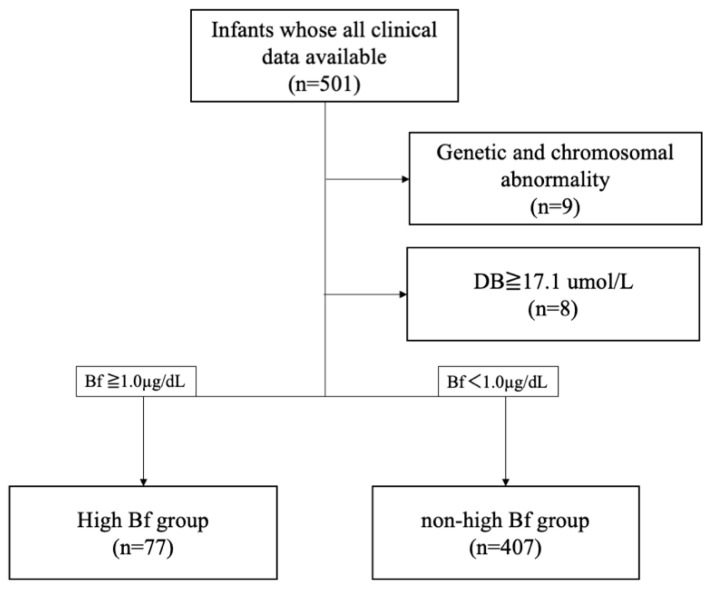
Patient enrollment. Bf, free bilirubin; DB direct bilirubin.

A total of 501 newborns were admitted to our institution between April 1, 2010 and December 31, 2018, and all required data, including *UGT1A1* variants, were available. Of these infants, nine were excluded due to genetic and chromosomal disorders (trisomy 21, *n* = 3; Kabuki syndrome, *n* = 1; Noonan syndrome, *n* = 1; Beckwith–Wiedemann syndrome, *n* = 1; hereditary spherocytosis, *n* = 3), and eight were excluded because their direct bilirubin (DB) levels at peak Bf timing were ≥ 1.0 mg/dL. Among the remaining 484 infants, 77 infants developed hyper-free bilirubinemia (High Bf group; Bf ≥ 1.0 µg/dL) and the other 407 infants did not (non-High Bf group; Bf < 1.0 µg/dL, Figure 1). 

Clinical characteristics of the enrolled infants are presented in Table 1. The number of cesarean sections, birth weight, gestational age, extremely preterm infants (less than 28 gestational weeks), Bf levels, TB levels, and DB levels were significantly higher, and the incidence of Apgar score at 1 min < 7 and Alb levels were significantly lower in the high Bf group than in the non-high Bf group. There was no significant difference in sex, incidence of Apgar scores at 5 min < 7, and age at the highest Bf.

### 3.1. UGT1A1 Variants

The incidence of two *UGT1A1* variants (rs4148323 and rs3064744) in the high Bf group and non-high Bf groups is shown in Table 2. The distribution of alleles (high Bf: G 70.8%, A 29.2%; non-high Bf: G 83.8%/A 16.2%; *p* < 0.001) and genotype frequencies of rs4148323 (high Bf: GG 54.5%, GA 32.5%, AA 13.0%; non-high Bf: GG 70.5%, GA 26.5%, AA 3.0%; *p* < 0.001) differed significantly between the high Bf and non-high Bf groups. The distribution of alleles (high Bf: (TA)_6_ 95.5%, (TA)_7_ 4.5%; non-high Bf: (TA)_6_ 88.0%, (TA)_7_ 12.0%; *p* < 0.01) and genotype frequencies of rs3064744 (high Bf: (TA)_6_(TA)_6_ 90.9%, (TA)_6_(TA)_7_ 9.1%, (TA)_7_(TA)_7_ 0%; non-high Bf: (TA)_6_(TA)_6_ 77.1%, (TA)_6_(TA)_7_ 21.6%, (TA)_7_(TA)_7_ 1.2%, *p* = 0.02) differed significantly between the high Bf and non-high Bf groups. Both variants were consistent with Hardy–Weinberg equilibrium (rs4148232: χ^2^ = 3.13, *p* = 0.077, rs3064744: χ^2^ = 0.10, *p* = 0.748).

### 3.2. Odds Ratio 

To determine the effect of *UGT1A1* variants on hyper-free bilirubinemia, we performed single-and multivariable logistic regression analyses (Table 3).

For rs4148323, the A allele was significantly associated with an increased risk of hyper-free bilirubinemia compared with the G allele (odds ratio (OR): 2.02, 95% confidence interval (CI): 1.38–2.98, *p* = 0.03). In addition, GA genotype (OR: 1.58, 95% CI: 0.92–2.72, *p* = 0.10), AA genotype (OR: 5.69, 95% CI: 2.32–14.0, *p* < 0.001), and GA and–AA genotype (OR: 1.99, 95% CI: 1.21–3.27, *p* < 0.01) carriers were at increased risk of hyper-free bilirubinemia compared to GG carriers. However, for rs3064744, the (TA)_7_ allele was significantly associated with a decreased risk of hyper-free bilirubinemia compared to the (TA)_6_ allele (OR: 0.34, 95% CI: 0.16–0.76, *p* < 0.01). In addition, (TA)_6_(TA)_7_ genotype (OR: 0.36, 95% CI: 0.16–0.80, *p* = 0.01) and (TA)_6_(TA)_7_ and (TA)(TA)_7_ genotype (OR: 0.34, 95% CI: 0.15–0.76, *p* < 0.01) carriers were at a decreased risk of hyper-free bilirubinemia compared to (TA)_6_(TA)_6_ genotype carriers. The OR of the (TA)_7_(TA)_7_ genotype could not be calculated because no (TA)_7_(TA)_7_ genotype carrier was present in the high Bf group. 

This tendency was maintained even after adjusting for confounding factors, including gestational age, sex, albumin levels, Apgar score at 1 min, and the other *UGT1A1* variant, in both rs4148323 (A allele over G allele [adjusted OR: 1.80, 95% CI: 1.19–2.72, *p* < 0.01]; GA genotype (adjusted OR: 1.29, 95% CI: 0.73–2.3, *p* = 0.39), AA genotype (adjusted OR: 5.15, 95% CI: 1.95–13.6, *p* < 0.001), and GA and AA genotype (adjusted OR: 1.66, 95% CI: 0.98–2.82, *p* = 0.06) carriers compared with GG carriers] and rs3064744 [(TA)_7_ allele over (TA)_6_ allele (adjusted OR: 0.42, 95% CI: 0.18–0.95, *p* = 0.04); (TA)_6_(TA)_7_ genotype (adjusted OR: 0.446, 95% CI: 0.192–1.04, *p* = 0.06) and (TA)_6_(TA)_7_&(TA)_7_(TA)_7_ genotype (adjusted OR: 0.417, 95% CI: 0.183–0.952, *p* = 0.04) carriers compared with (TA)_6_(TA)_6_ genotype carriers].

## 4. Discussion

This study is the first to identify the incidence of *UGT1A1* variants rs4148323 and rs3064744 in newborns with hyper-free bilirubinemia. In addition, we found that the A allele in rs4148323 was a significant risk factor, whereas the (TA)_7_ allele in rs3064744 was a significant protective factor for developing hyper-free bilirubinemia.

In this study, the allele frequency was 16% for the A allele in rs4148323 and 12% for the (TA)_7_ allele in rs3064744 in the non-high Bf group, which are consistent with frequencies in the Japanese population in the Japanese Multiple Genome Database (jMorp; 18% and 11%, respectively) [35], suggesting that the participants were not genetically biased. In contrast, the allele frequencies in the high Bf group were 29% and 4%, respectively, suggesting that the A allele in rs4148323 was a risk factor and the (TA)_7_ allele in rs3064744 was a protective factor for developing high hyper-free bilirubinemia. In addition, we did not find any infants with rs4148323/rs3064744 AA/(TA)_6_(TA)_7_, GA/(TA)_7_(TA)_7_, or AA/(TA)_7_(TA)_7_ genotype (Supplementary Table S1). In a previous study including 42 infants with hyper-total bilirubinemia and 50 controls, no cases with these three genotypes were reported [36]. This might be because rs4148323 and rs3064744 were in a linkage disequilibrium state, and thus, there were no alleles in which these two variants coexisted.

To date, several known risk factors have been shown to increase serum Bf levels. First, Bf is indirect bilirubin that is not bound to albumin; thus, low serum albumin levels can be a risk factor for hyper-free bilirubinemia [13]. Preterm birth also poses a risk of hyper-free bilirubinemia because of altered bilirubin–albumin binding in preterm infants, which is due to low and variable bilirubin binding capacity and affinity in these infants [37]. In addition, free fatty acids, antibiotic use, ibuprofen use, infection, hypothermia, hypoxia, acidosis, hypercapnia, and asphyxia, which compete for bilirubin-albumin binding, are also known risk factors [38]. However, there are no reports regarding genetic factors involved in hyper-free bilirubinemia. In this study, we showed for the first time that two major genetic variants of *UGT1A1* act as risk and protective factors for the development of hyper-free bilirubinemia, even after adjustments with known risk factors.

In this study, we showed that the A allele in rs4148323 is a significant risk factor for developing hyper-free bilirubinemia. It has been reported that the A allele in rs4148323 is common in East Asian regions, with allele frequencies of 0.13 in Japan and 0.23 in South Korea and China [36]; this is comparable to its frequency in the non-high Bf group in our study (0.16). In addition, it has been reported that this variant is a risk factor for hyper-total bilirubinemia in Asia [15,16,17,18,19]. Notably, a study that utilized liver biopsy specimens showed that patients with this heterozygous variant had <50% normal hepatic bilirubin UDP-glucuronosyltransferase activity [39]. Our results indicate that the A allele in rs4148323 is a direct risk factor for hyper-free bilirubinemia; therefore, we believe that this variant is an important risk factor for the development of bilirubin encephalopathy.

In contrast, the (TA)_7_ allele in rs3064744 is a protective factor against hyper-free bilirubinemia. Regarding this variant, there have been several reports from outside Asia that it is a risk factor for neonatal jaundice [22,23], and there are reports from Asia that suggest that it is a protective factor [15,20,21]. Thus, its role remain controversial. In a previous meta-analysis regarding the association of neonatal hyperbilirubinemia and UGT1A1 gene polymorphisms, ethnic heterogeneity was found in th (TA)7 variant between Caucasians and Asians. In a subgroup analysis of that study, (TA)7 was significantly associated with hyperbilirubinemia in Caucasians but not in Asians. In particular, a subgroup analysis that focused on three Japanese studies indicated that (TA)7 was associated with a significant reduction in hyperbilirubinemia [40]. The present study is the first to report that this variant has a protective effect on hyper-free bilirubinemia, and this finding is consistent with previous reports from Asia. The reason why the effect of this variant on neonatal jaundice differs depending on race has not been clarified; however, a similar phenomenon has been observed for other diseases, in which the action of a variant differs depending on race. For example, VEGF c.-94C>T in chronic immune-mediated inflammatory disease functions as a protective factor in Asian populations and a risk factor in Caucasian populations [41], or CPB2 c.505G>A in venous thrombosis functions as a risk factor in Asians and a protective factor in Caucasian populations [42]. It is speculated that these differences in action may be caused by other genes, interactions between genes, and environmental factors, rather than the direct function of the detected variants [41,43]. Considering only the two variants examined in this study, few patients had both variants in a heterozygous presentation, none had one variant homozygous and the other variant heterozygous or both variants homozygous. Thus, we speculated that the (TA)_7_ allele in rs3064744 does not directly affect the phenotype, but that the variants linked by the presence or absence of the (TA)_7_ allele in rs3064744 differ between races, which might cause interracial differences.

This study had several limitations. First, since this was a retrospective study, *UGT1A1* variants could only be determined in cases with residual blood samples; thus, we could not perform genotyping for all cases. Therefore, there is a possibility of selection bias. In the future, we intend to conduct a prospective study of all admitted infants using noninvasively collected specimens, such as umbilical cord blood and tissues. Second, in addition to the two *UGT1A1* variants, several other *UGT1A1* variants and variants of other genes, such as *SLCO1B1*, *G6PD*, and *SLCO1B3*, were shown to be associated with neonatal hyper-total bilirubinemia in a genome-wide association study [44,45]. Thus, in the future, it is necessary to investigate the true relationship between *UGT1A1* variants and hyper-free bilirubinemia by examining other related genes. Third, we adjusted for gestational age, albumin levels, and asphyxia, which are known to cause hyper-free bilirubinemia; however, we could not examine the effect of medications that might affect Bf levels or UGT1A1 activity. Therefore, future studies including these factors are needed. Finally, since Bf levels cannot be measured in daily clinical practice in countries other than Japan, the reference values for Bf have not been clearly determined worldwide. Therefore, it remains unclear whether the definition of high Bf (Bf ≥ 1.0 µg/dL) used in this study reflects the increased risk of development of kernicterus. We await the results of an ongoing multicenter prospective cohort study that aims to examine treatment criteria, including Bf measurement, for infants with hyperbilirubinemia [46].

## 5. Conclusions

This study is the first to show that the A allele in rs4148323 is a risk factor and that the (TA)_7_ allele in rs3064744 is a protective factor for developing hyper-free bilirubinemia in Japanese newborns.

## Figures and Tables

**Table 1:** Clinical Characteristics.

	High Bf (*n* = 77)	Non-High Bf (*n* = 407)	*p*-Value
Male	50 (64.9%)	226 (55.5%)	0.134
Cesarean section	29 (37.7%)	258 (63.4%)	<0.001
Birth weight (g)	2622 (320, 4284)	2076 (1036, 4732)	<0.001
Gestational week (weeks)	36 (22, 41)	34 (26, 41)	0.008
Eextremely preterm infants (less than 28 gestational weeks)	2 (2.6%)	47 (11.5%)	0.013
Apgar score at 1 min < 7	10 (13.0%)	105 (25.8%)	0.019
Apgar score at 5 min < 7	2 (2.6%)	28 (6.9%)	0.200
Highest Bf (µg/dL)	1.04 (1.00, 1.90)	0.57 (0.02, 0.99)	<0.001
Age at the highest Bf (day)	4 (1, 39)	3 (1, 39)	0.086
T-bil at the highest Bf (μmol/L)	289 (181.3, 427.5)	188.1 (35.9, 350.6)	<0.001
D-bil at the highest Bf (μmol/L)	5.1 (1.7, 5.1)	3.4 (1.7, 5.1)	<0.001
Alb at the highest Bf (g/dL)	3.2 (2.4, 4.2)	3.4 (1.9, 4.6)	0.006

Bf, free bilirubin; T-bil, total bilirubin; D-bil, direct bilirubin; Alb, albumin. Data are expressed as number (%) or median (range).

**Table 2:** Genotype and Allele Counts.

	High Bf	Non-High Bf	*p*-Value
rs4148323			
GG	42 (54.5%)	287 (70.5%)	
GA	25 (32.5%)	108 (26.5%)	
AA	10 (13.0%)	12 (2.95%)	<0.001
G allele	109 (70.8%)	682 (83.8%)	
A allele	45 (29.2%)	132 (16.2%)	<0.001
rs3064744			
(TA)_6_/(TA)_6_	70 (90.9%)	314 (77.1%)	
(TA)_6_/(TA)_7_	7 (9.1%)	88 (21.6%)	
(TA)_7_/(TA)_7_	0 (0%)	5 (1.2%)	0.017
(TA)_6_ allele	147 (95.5%)	716 (88.0%)	
(TA)_7_ allele	7 (4.5%)	98 (12.0%)	0.044

Bf, free bilirubin. Data are expressed as number (%).

**Table 3:** Multivariable Logistic Regression Analysis.

Factor	High Bf (*n* = 77)	Non-High Bf (*n* = 407)	OR (95% CI)	*p*	Adjusted OR (95% CI)	*p*
rs4148323						
GG	42 (54.5%)	287 (70.5%)	1		1	
GA	25 (32.5%)	108 (26.5%)	1.58 (0.92–2.72)	0.098	1.29 (0.725–2.3)	0.385
AA	10 (13.0%)	12 (2.95%)	5.69 (2.32–14.0)	<0.001	5.15 (1.95–13.6)	<0.001
GA and AA	35 (45.5%)	120 (29.5%)	1.99 (1.21–3.27)	0.006	1.66 (0.980–2.82)	0.060
G	109 (70.8%)	682 (83.8%)	1		1	
A	45 (29.2%)	132 (16.2%)	2.02 (1.38–2.98)	0.034	1.80 (1.19–2.72)	0.005
rs3064744						
(TA)_6_(TA)_6_	70 (90.9%)	314 (77.1%)	1		1	
(TA)_6_(TA)_7_	7 (9.1%)	88 (21.6%)	0.357 (0.158–0.804)	0.013	0.446 (0.192–1.04)	0.061
(TA)_7_(TA)_7_	0 (0%)	5 (1.2%)	N.D		N.D	
(TA)_6_(TA)_7_ and (TA)_7_(TA)_7_	7 (9.1%)	96 (22.9%)	0.338 (0.150–0.759)	0.009	0.426 (0183–0.989)	0.047
(TA)_6_	147 (95.5%)	716 (88.0%)	1		1	
(TA)_7_	7 (4.5%)	98 (12.0%)	0.342 (0.155–0.757)	0.008	0.417 (0.183–0.952)	0.038

Bf, free bilirubin; OR, odds ratio; N.D, no data. Adjusted OR were obtained from a logistic regression analysis, after adjusting for gestational age, sex, albumin levels, Apgar score at 1 min, and the other *UGT1A1* variant. Data are expressed as number (%) or odds ratio (95% CI).

## Data Availability

All data generated or analyzed during this study are included in this published article and its supplementary information file.

## Supplementary Materials

The following supporting information can be downloaded at: https://www.mdpi.com/article/10.3390/ijerph192013090/s1, Table S1: Genotype of rs4148323/rs3064744 for enrolled infants.
