# Supplementary material for: Influence of UGT1A1 Genetic Variants on Free Bilirubin Levels in Japanese Newborns: A Preliminary Study
Source: Int J Environ Res Public Health. 2022 Oct 12;19(20):13090. doi: 10.3390/ijerph192013090 (PMC9603041; doi:10.3390/ijerph192013090)
Supplement: Supplementary file 1 [file ijerph-19-13090-s001.zip › ijerph-1939683-supplementary.pdf]

Supplementary Table S1. Genotype of rs4148323/rs3064744 for enrolled infants

	rs4148323/rs3064744	GG	GA	AA
All enrolled infants (n=484)	**$(TA)_6/(TA)_6$**	251 (51.9%)	111 (22.9%)	22 (4.5%)
	**$(TA)_6/(TA)_7$**	73 (15.1%)	22 (4.5%)	0 (0%)
	**$(TA)_7/(TA)_7$**	5 (1.0%)	0 (0%)	0 (0%)
High Bf (n=77)	**$(TA)_6/(TA)_6$**	37 (48.1%)	23 (29.9%)	10 (13.0%)
	**$(TA)_6/(TA)_7$**	5 (6.5%)	2 (2.6%)	0 (0%)
	**$(TA)_7/(TA)_7$**	0 (0%)	0 (0%)	0 (0%)
non-high Bf (n=407)	**$(TA)_6/(TA)_6$**	214 (52.3%)	88 (21.6%)	12 (2.9%)
	**$(TA)_6/(TA)_7$**	68 (16.7%)	20 (4.9%)	0 (0%)
	**$(TA)_7/(TA)_7$**	5 (1.2%)	0 (0%)	0 (0%)

UB, unbound bilirubin
